# Two different clinical forms of squamous cell carcinoma (SCC) in the perineum and vulva of two mares

**DOI:** 10.1186/s12917-020-02678-9

**Published:** 2020-11-30

**Authors:** Andrzej Raś, Iwona Otrocka-Domagała, Małgorzata Raś-Noryńska

**Affiliations:** 1 Department of Animal Reproduction with Clinic, Faculty of Veterinary Medicine, University of Warmia and Mazury in Olsztyn, ul. Oczapowskiego 14, 10-781 Olsztyn, Poland; 2 Department of Pathological Anatomy, Faculty of Veterinary Medicine, University of Warmia and Mazury in Olsztyn, ul. Oczapowskiego 13, 10-781 Olsztyn, Poland; 3 Department of Parasitology and Invasive Diseases, Faculty of Veterinary Medicine, University of Warmia and Mazury in Olsztyn, ul. Oczapowskiego 13, 10-781 Olsztyn, Poland

**Keywords:** Mare, Perineum, Squamous cell carcinoma

## Abstract

**Background:**

Genital malignant neoplasms in mares are relatively rare. The treatment involve surgical removal of the tumour masses, chemotherapy or both.

**Case presentation:**

Two elderly warmblood mares, aged 16 and 20 were presented in University Clinic with the lumpy lesions at the region of perineum and left labia. Surgical removals of tumour masses were performed on standing animals. Removed tissues were subjected to histopathological examination which confirmed SCC.

**Conclusions:**

Clinical and ultrasound examination of reproductive organs in both mares showed no inflammatory or neoplastic changes. Both mares healed within 2 weeks after surgery and showed no signs of tumour recurrence for the following year despite no chemotherapy treatment.

## Background

Genital neoplasms in female animals are quite rare, except for leiomyoma in cows and bitches, fibropapilloma in cows and venereal tumors in bitches.

Squamous cell carcinoma (SCC) is a malignant tumor of non-glandular epithelial tissue, composed of large epithelial cells, showing the presence of intercellular bridges and the production of keratin, occurring in the form of pearls or single keratinized cells. It is a fairly rare malignant tumor in horses that appears on the skin, on the border between the skin and mucosa, and on non-pigmented mucous membranes. It is more common on the penis and prepuce in older stallions and geldings, but it has also been described in horses in the nasal cavity, mouth, larynx, nasal sinuses, vulva and vagina [1]. Most of the tumors in mares form cauliflower-shape, solid masses on the border of the skin and mucous membrane of the labia with a tendency to proliferate and metastasize to the superficial and deep inguinal lymph nodes, and to the lungs [2]. Tumors located in the ano-genital area often bleed, ulcerate and spontaneously break down, giving an unpleasant odor. These neoplasms are referred to as the second most common in horses (after sarcoids) [3].

## Case presentation

**Fig. 1:**
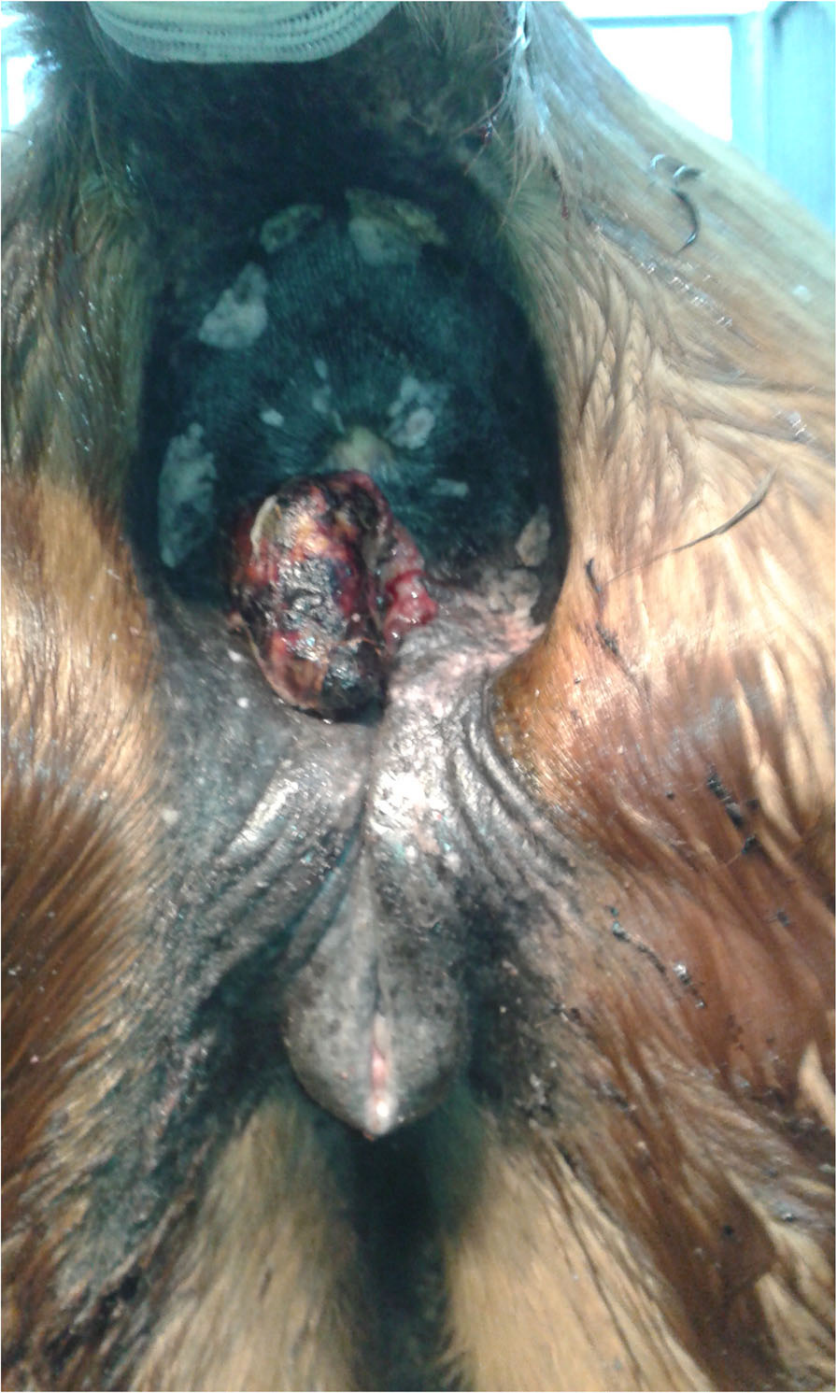
Clinical presentation of the tumor by mare XX

A thoroughbred mare (XX), 20 years old, in medium condition, belonging to the Foundation taking care of old horses, was reported to the Clinic of Department of Animal Reproduction, Faculty of Veterinary Medicine, University of Warmia and Mazury in Olsztyn. The carers of the mare noticed a lumpy lesion in the area of the left labia and perineum, peduncled, fairly well demarcated from healthy tissue, which often bled and gave an unpleasant smell (Fig. 1).

**Fig. 2:**
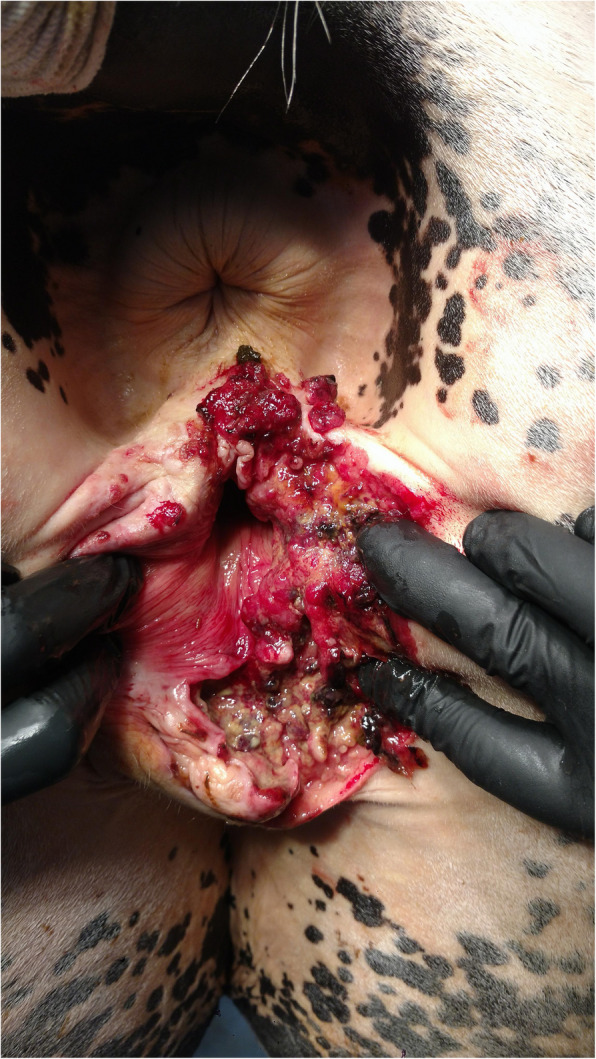
Clinical presentation of the tumor by mare SP

The second mare reported as a patient to the Clinic was 16 years old, SP breed, in very good condition, gray, and showed degenerative and proliferative changes in the unpigmented skin of the left labia and clitoris, reaching into the vaginal vestibule and deep into soft tissues (Fig. 2).

Both mares underwent palpation of tumor nodules, clinical and ultrasound examination of nearby lymph nodes, and examination of the reproductive organ. Internal reproductive organs in both mares showed no inflammatory or neoplastic changes. Both mares were in seasonal anoestrus. Inguinal lymph nodes were without inflammatory changes and enlargement features. Haematological blood parameters in both mares were within the physiological norms (mare XX - WBC 10.8 thousand / ml, RBC 5.7 million / ml, Hb - 10.8%, Htc - 40.2%; mare SP - WBC 9.2 thousand / ml, RBC 5.9 million / ml, Hb - 11.2%, Htc - 44.3%).

In both cases, surgical removal of neoplastic masses was performed on a standing animal. For anesthesia, detomidine (Domosedan-Orion) and butorphanol (Butomidor-Orion) were used and epidural anesthesia (2% Polocaine-Polfa) was performed. The nodular tissues were removed with the unchanged tissue margin, and then plastic correction of the vulva and perineum was performed using the modified Caslik method [4]. Two layers of sutures were placed using absorbable braided thread (Safil 1- Braun). The stitched wounds were protected with chlortetracycline spray (CTC -spray- Dechra) + trance ointment.

After excision, both tumors were measured and weighed, cut into smaller parts, fixed in 10% neutral buffered formalin, processed by the routine paraffin technique, and stained with hematoxylin and eosin (HE) for histopathological examination.

The cauliflower- shape tumor, removed from the left labia and perineum of the XX mare, was 8 × 5 cm long and weighed 167 g. Its surface was irregular and ulcerated. The lesion removed from the SP mare weighed 345 g, had an irregular, tumor-proliferative shape, penetrating deep into the surrounding tissues.

**Fig. 3:**
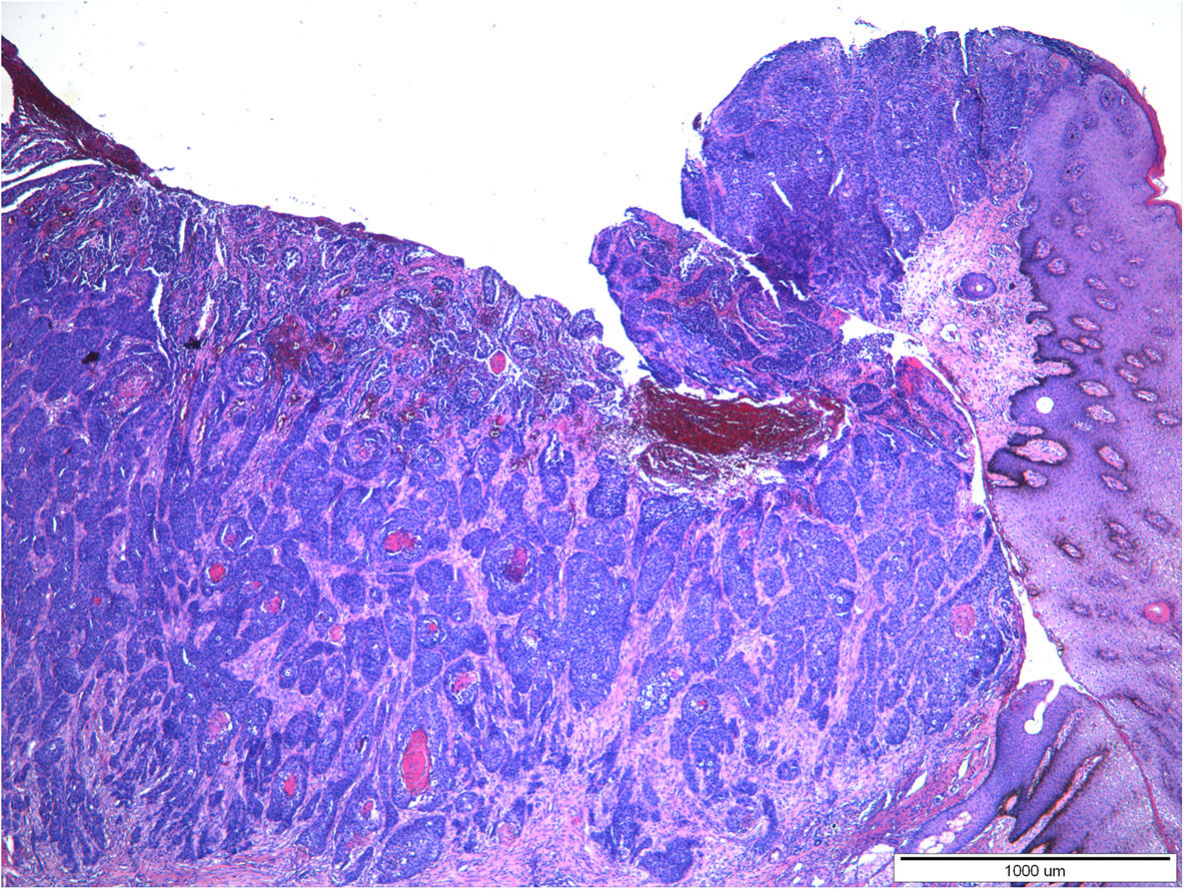
A 20-year-old mare, moderately-differentiated squamous cell carcinoma (SCC grade 3) of the vulva. Locally invasive, unencapsulated tumor, having an association with overlying epidermis. Neoplastic cells form nests, cords and trabeculae with occasionally observed parakeratosis or keratin pearls formation. HE. Magnification 4x. (klacz – badanie H/17/407–408)

**Fig. 4:**
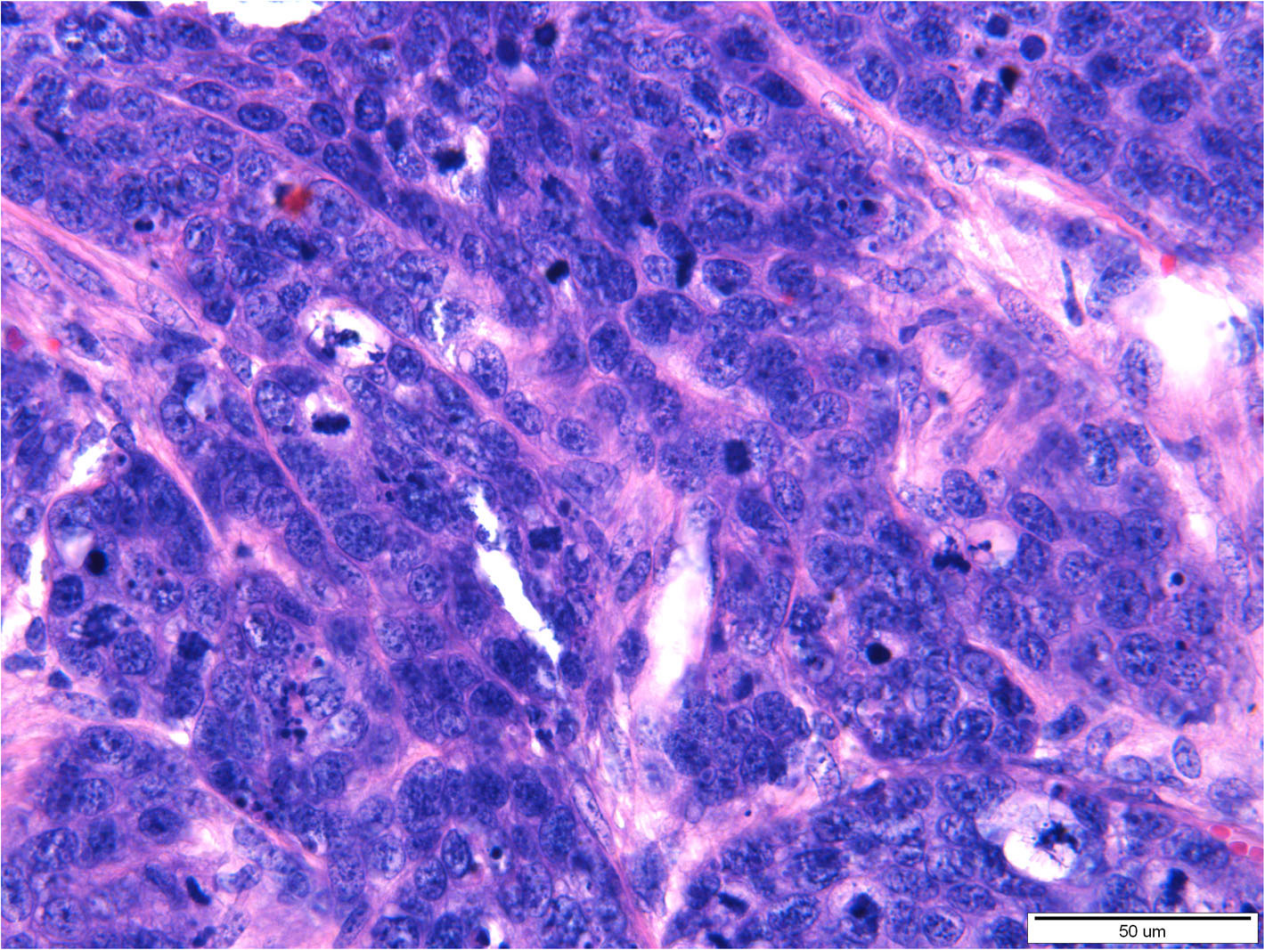
A 20-year-old mare, moderately-differentiated squamous cell carcinoma (SCC grade 3) of the vulva. Moderately- and poorly-differentiated, polygonal, round to oval neoplastic cells form nests, cords, and trabeculae. Anisocytosis and anisokaryosis of neoplastic cells are marked. Numerous mitotic figures are visible. Magnification 60x. (klacz – badanie H/17/407–408)

**Fig. 5:**
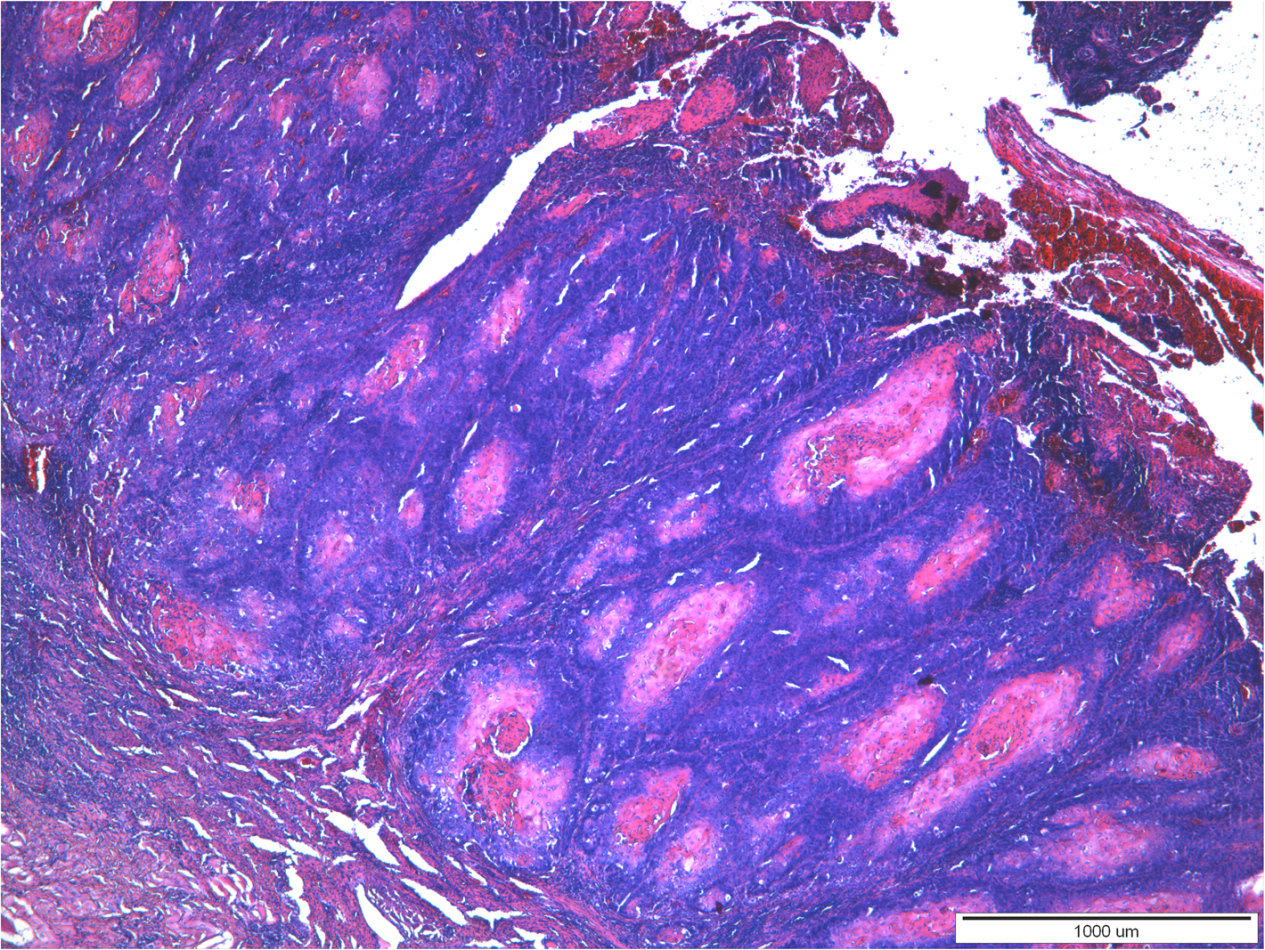
A 16-year-old mare, moderately-differentiated squamous cell carcinoma (SCC grade 3) of the vulva. Locally invasive, unencapsulated, ulcerated tumor is seen. Neoplastic cells form nests with quite often observed parakeratosis or keratin pearls formation. HE. Magnification 4x. (klacz – badanie H/18/5927)

**Fig. 6:**
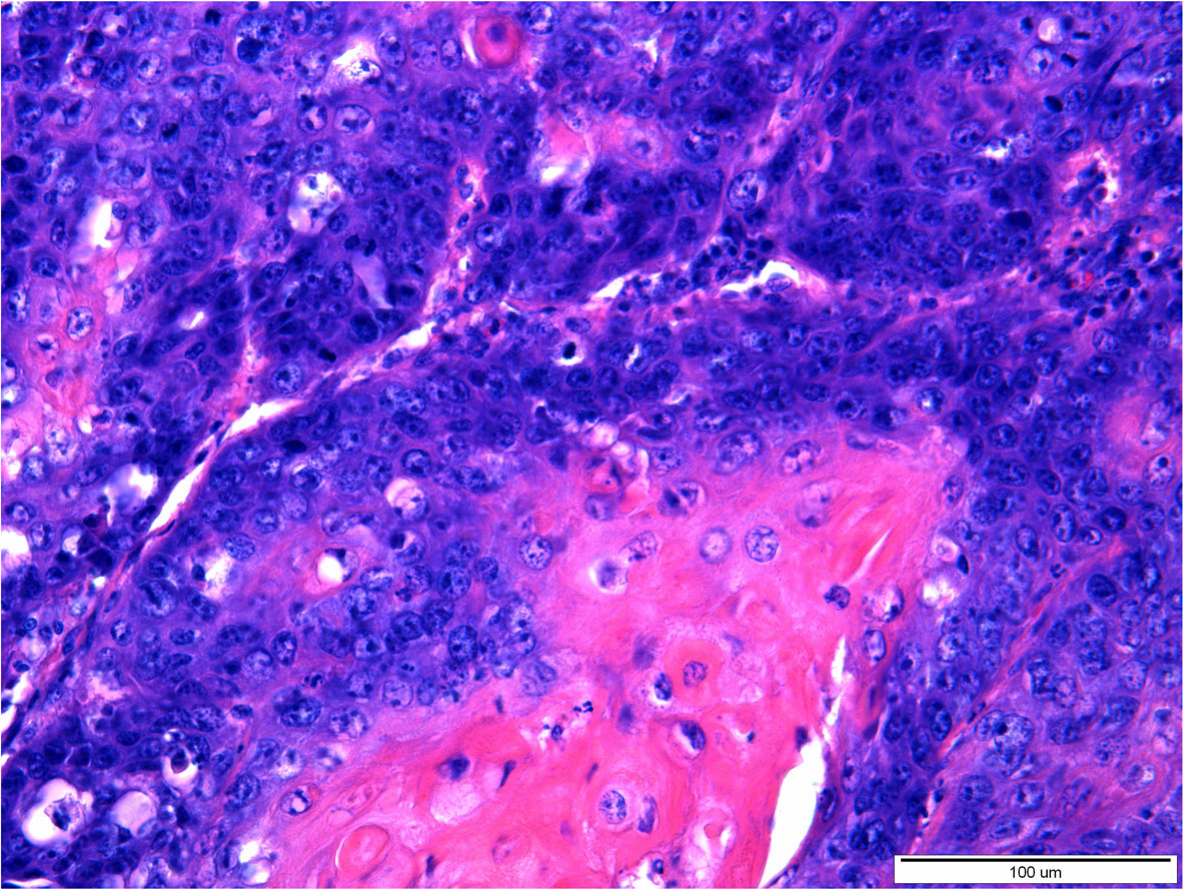
A 16-year-old mare, moderately-differentiated squamous cell carcinoma (SCC grade 3) of the vulva. Moderately-differentiated, polygonal, round to oval neoplastic cells form nests separated by scant stroma. Anisocytosis and anisokaryosis of neoplastic cells are marked. Parakeratosis of single cells or groups of cells is visible. Magnification 40x. (klacz – badanie H/18/5927)

Histopathological examination of both tumors revealed the presence of a poorly demarcated, unencapsulated, multifocal, and diffuse tumors originating from a stratified squamous epithelium, localized in the superficial, middle, and deep layers of the skin. Both lesions were ulcerated. Neoplastic cells formed anastomosing nests, cords, and trabeculae supported by desmoplastic stroma with occasionally observed parakeratosis or keratin pearls formation. Necrosis was sometimes present in the center of the tumor nests. Neoplastic cells were polygonal, round to oval with pleomorphic nuclei, unevenly dispersed chromatin, and 1–5 often large in size nucleoli. Anisocytosis and anisokaryosis were high. The mitotic rate averages 8–15 per 40x HPF. In both tumors, neoplastic cells were located close to the lymphatic vessels. Based on histopathological examination squamous cell carcinoma (SCC) of grade-III was diagnosed in both lesions (Figs. 3, 4, 5 and 6).

Healing of postoperative wounds in both mares was monitored daily and protected antibacterially. After about 2 weeks, the sutures were removed and no disturbing changes suggesting recurrence were observed. Both mares were observed at the owners for a year. They were in good condition with no signs of recurrence.

## Discussion and conclusion

According to Taylor and Haldorson [5], only about 13% of SCC cases in horses involve external genitalia. When occurring at the vulva, they usually take a proliferative form, penetrating deep into the surrounding tissues, reaching into the vaginal vestibule [6]. Cases of lesions located on the skin of the labia usually have the form of demarcated, ulcerative tumors [7]. The reasons for these changes should be seen in chronic irritation, prolonged exposure of unpigmented skin to UV rays, and viral infections [8]. There is practically no developed effective prevention of such changes in horses. In the case of gray horses with unpigmented skin and mucous membranes, protection against exposure to UV rays is suggested. In addition to surgical treatment, various authors recommend supplementing it with chemotherapy, which, however, is not always possible due to the cost and availability of drugs. In such a situation, appropriate resection of lesions and post-operative monitoring carried out jointly by the caregiver and periodic veterinary examination allow for the assessment of the effectiveness of surgical treatment and early detection of any possible recurrence.

## Data Availability

The datasets used and/or analysed during the current study are available from the corresponding author on reasonable request.

## Abbreviations

SCC: Squamous Cell Carcinoma
WBC: White Cell Count
RBC: Red Cell Count
Hb: Hemoglobin
Htc: Hematocrit
SP breed: Noble half-blood
